# A nationwide assessment of perceptions of research-intense academic careers among predoctoral MD and MD-PhD trainees

**DOI:** 10.1017/cts.2020.18

**Published:** 2020-03-04

**Authors:** Jennifer M. Kwan, Omar Toubat, Andrew M. Harrison, Megan Riddle, Brian Wu, Hajwa Kim, David W. Basta, Alexander J. Adami, Dania Daye

**Affiliations:** 1 Section of Cardiovascular Medicine, Yale University School of Medicine, New Haven, CT, USA; 2 American Physician Scientists Association (APSA), Westford, MA, USA; 3 Keck School of Medicine of the University of Southern California, Los Angeles, CA, USA; 4 Division of Pulmonary and Critical Care Medicine, Mayo Clinic, Rochester, MN, USA; 5 Department of Psychiatry, University of Washington, Seattle, WA, USA; 6 Center for Clinical and Translational Science, University of Illinois at Chicago, Chicago, IL, USA; 7 Department of Radiology, Massachusetts General Hospital, Boston, MA, USA

**Keywords:** Physician-scientists, academic medicine, translational research, basic science research, clinical research, disparities

## Abstract

**Introduction:**

While previous studies have described career outcomes of physician-scientist trainees after graduation, trainee perceptions of research-intensive career pathways remain unclear. This study sought to identify the perceived interests, factors, and challenges associated with academic and research careers among predoctoral MD trainees, MD trainees with research-intense (>50%) career intentions (MD-RI), and MD-PhD trainees.

**Methods:**

A 70-question survey was administered to 16,418 trainees at 32 academic medical centers from September 2012 to December 2014. MD vs. MD-RI (>50% research intentions) vs. MD-PhD trainee responses were compared by chi-square tests. Multivariate logistic regression analyses were performed to identify variables associated with academic and research career intentions.

**Results:**

There were 4433 respondents (27% response rate), including 2625 MD (64%), 653 MD-RI (15%), and 856 MD-PhD (21%) trainees. MD-PhDs were most interested in pursuing academia (85.8%), followed by MD-RIs (57.3%) and MDs (31.2%). Translational research was the primary career intention for MD-PhD trainees (42.9%). Clinical duties were the primary career intention for MD-RIs (51.9%) and MDs (84.2%). While 39.8% of MD-PhD respondents identified opportunities for research as the most important career selection factor, only 12.9% of MD-RI and 0.5% of MD respondents shared this perspective. Interest in basic research, translational research, clinical research, education, and the ability to identify a mentor were each independently associated with academic career intentions by multivariate regression.

**Conclusions:**

Predoctoral MD, MD-RI, and MD-PhD trainees are unique cohorts with different perceptions and interests toward academic and research careers. Understanding these differences may help to guide efforts to mentor the next generation of physician-scientists.

## Introduction

### The Declining Physician-Scientist Workforce

Physician-scientists are an important part of the academic medical enterprise. Combining skills and knowledge from medical and scientific training, physician-scientists are uniquely positioned to advance academic medicine by answering critical scientific questions related to human health and disease [1,2]. Despite this importance, the number of physician-scientists has been declining for decades [3–5]. Recognition of this decline has spurred a number of groups to examine how to rejuvenate physician-scientist training [6].

Recognition of diminishing physician-scientist participation in academic medical research is not new. An assessment of National Institutes of Health (NIH) grant applications and awards through the early 2000s revealed reduced success among MD applicants despite a stable number of grant applications from this group [7]. MD participation in NIH-funded medical research peaked in the late 1980s. This decline was interrupted only temporarily when the NIH budget was doubled at the start of the 21st century [8]. By 2011, the most dramatic decline in grants awarded to physician-scientists was MD applicants. MD-PhD applicants began to decline after a steady trend of increasing participation. Factors contributing to these changes include poor funding environment, competition, pressures for clinical productivity, lack of opportunity for professional advancement, and poor work–life balance [9–11]. To combat faculty attrition, a number of innovative institutions have developed programs including structured mentorship and earlier intervention by leadership [12–14]. However, these important interventions occur after the transition from trainee to junior faculty [15]. This has resulted in a failure to determine and address factors influencing attrition prior to attaining a faculty position. Understanding the earliest stages of training is one approach to combat this decline in physician-scientists.

### Trainee Career Choices Beyond a Dual-Degree

Training pathways and career choices pursued prior to attaining a first faculty position have been investigated. Outcomes of MD-PhD training programs have been examined retrospectively by several groups to determine the trajectories of trainee careers [16,17]. An important and encouraging result of these studies is the high proportion of physician-scientists, including MSTP trainees, who choose to remain in academic medicine. However, these studies often do not examine how or why trainees seek and obtain faculty positions.

### The Need for Further Investigation

Several recent studies have described the career trajectories and outcomes of MD-PhD program graduates [18–22]. However, there is less information on the attitudes, perceived obstacles, and career perspectives of current trainees. Much of the existing literature on trainee perspectives has been limited by focusing only on MD-PhD trainee cohorts at individual institutions [23–25]. Single-degree investigators are a significant and largely understudied proportion of the physician-scientist workforce; thus, an assessment of this trainee population is a necessity.

To this end, the central objective of the present study was to examine the factors associated with academic and research career interests in a nationally representative cohort of single- and dual-degree predoctoral physician-scientist trainees. This objective was pursued using a previously validated survey employed in a pilot study of a similar trainee population [26]. We hypothesized that MD, DO, and MD/DO-PhDs are unique cohorts with different perceptions and interests toward academic and research careers.

## Methods

### Study Design

This study was reviewed and exempted by the Institutional Review Boards at the University of Illinois at Chicago and University of Pennsylvania.

In this analysis, MD refers to both MD and DO trainees. MD-RI refers to both MD and DO trainees with research-intense career plans. MD-RI status was defined by a self-reported career interest of at least 50% research and was not intended to identify trainees enrolled in formal research pathway programs or curricula. This minimum research interest was selected because 50% is often the contractual maximum research to clinical ratio for surgeon-scientists [27]. MD-PhD refers to both MD-PhD and DO-PhD trainees.

### Data Collection

A 70-item survey (Supplementary information) was designed with feedback from a survey design team at the University of Illinois at Chicago, as well as lessons learned from a pilot study using this survey at a group of representative American medical schools [26]. This survey is a validated instrument with internal reliability to assess factors deemed important by trainees for their future careers and anticipated career challenges. The survey was sent to 32 nationally representative institutions via representatives of the American Physician Scientists Association (APSA) and the American Medical Women’s Association. Survey response variables, including answer choices for career intentions, career sector, types of research (basic vs. translational vs. clinical), and obstacles, were not defined for the respondents and were, therefore, left to their interpretation.

Data were collected using an online survey tool (SurveyMonkey, San Mateo, CA). The survey was sent in September 2012 via email to all MD and MD-PhD trainees at these universities through trainee list serves and institutional representatives of APSA. One osteopathic medical school with DO and DO-PhD trainees was included in this study. Data collection ended in December 2014. Participants had the option to enter an institutional email address for a randomly selected $50 Amazon gift certificate. Email addresses were kept separate from survey responses to maintain anonymity of responses.

### Statistical Analysis

Survey results were analyzed to identify significant differences in perceptions of factors influencing career interests between MD, MD-RI, and MD-PhD trainees. Chi-squared tests were used to measure associations between categorical variables. When data violated minimum expected cell counts, Fisher’s exact test was performed. Logistic regression was used to identify factors associated with the intention to pursue academic and research careers. All tests were performed using SPSS and were two-sided with *p* < 0.05 considered statistically significant.

## Results

### Demographics

There were 4433 respondents to the survey, yielding a 27% response rate. Demographic characteristics of respondents segregated by MD, MD-RI, and MD-PhD training status are summarized in Table 1. Overall, there were significantly more female than male respondents (56.3% female). However, there were significantly fewer female than male MD-PhD respondents (47.6%). Although trainees from each stage of medical and graduate training were represented, first-year medical students comprised the largest contingent of respondents (28.2%).

**Table 1.** Demographic characteristics of respondents by MD, MD-RI, and MD-PhD

Demographic	Total, *n* (%)	MD-PhD, *n* (%)	MD-RI, *n* (%)	MD, *n* (%)	*p*-value
Gender					<.0001
Female	2328 (56.3%)	394 (47.6%)	366 (56.1%)	1568 (59.7%)	
Male	1795 (43.4%)	459 (52.4%)	284 (43.5%)	1052 (40.1%)	
Other	11 (0.27%)	3 (0.35%)	3 (0.46%)	5 (0.19%)	
Total	4134 (100%)	856 (100%)	653 (100%)	2625 (100%)	
Training stage					<.0001
Medical school year 1	1170 (28.2%)	159 (18.6%)	204 (31.1%)	807 (30.7%)	
Medical school year 2	1046 (25.2%)	131 (15.3%)	160 (24.4%)	755 (28.7%)	
Medical school year 3	680 (16.4%)	68 (7.94%)	116 (17.7%)	496 (18.8%)	
Medical school year 4	685 (16.5%)	84 (9.81%)	120 (18.3%)	481 (18.3%)	
Graduate school year 1	133 (3.21%)	114 (13.3%)	5 (0.76%)	14 (0.53%)	
Graduate school year 2	103 (2.48%)	96 (11.2%)	4 (0.61%)	3 (0.11%)	
Graduate school year 3	85 (2.05%)	80 (9.35%)	1 (0.15%)	4 (0.15%)	
Graduate school year 4	88 (2.12%)	82 (9.58%)	1 (0.15%)	5 (0.19%)	
Graduate school year 5 or more	35 (0.84%)	32 (3.74%)	2 (0.30%)	1 (0.04%)	
Total	4145 (100%)	856 (100%)	656 (100%)	2633 (100%)	
Race					<.0001
White	2862 (71.0%)	592 (71.5%)	374 (58.5%)	1896 (73.9%)	
Black or African American	167 (4.14%)	30 (3.62%)	35 (5.48%)	102 (3.98%)	
American Indian or Alaska Native	10 (0.25%)	1 (0.12%)	0 (--)	9 (0.35%)	
Asian or Pacific Islander	427 (10.6%)	101 (12.2%)	89 (13.9%)	237 (9.24%)	
Multiracial or other	567 (14.1%)	104 (12.6%)	141 (22.1%)	322 (12.6%)	
Total	4033 (100%)	828 (100%)	182 (100%)	638 (100%)	
Ethnicity					0.0075
Hispanic	242 (5.88%)	45 (5.29%)	56 (8.51%)	141 (5.41%)	
Not Hispanic	3871 (94.1%)	805 (94.7%)	602 (91.5%)	2464 (94.6%)	
Total	4113 (100%)	850 (100%)	658 (100%)	2605 (100%)	
Marital status					<.0001
Is married/partnered	1055 (26.1%)	276 (32.8%)	129 (19.6%)	650 (25.6%)	
Is *not* married/partnered	2987 (73.9%)	566 (67.2%)	529 (80.4%)	1892 (74.4%)	
Total	4042 (100%)	842 (100%)	658(100%)	2542 (100%)	
Parental status					0.0024
Has a child/children	231 (5.72%)	68 (8.08%)	28 (4.26%)	135 (5.31%)	
Does *not* have a child/children	3810 (94.3 %)	774 (91.9%)	629 (95.7%)	2407 (94.7%)	
Total	4041 (100%)	842 (100%)	657 (100%)	2542 (100%)	
How primarily paid for medical school					
MD-PhD or DO-PhD sponsored	751 (18.4%)	749 (88.2%)	1 (0.15%)	1 (0.04%)	<.0001
Scholarships	384 (9.43%)	11 (1.30%)	93 (14.2%)	280 (10.9%)	<.0001
Grants	75 (1.84%)	5 (0.59%)	12 (1.84%)	58 (2.26%)	<.0001
Loans	2132 (52.4%)	62 (7.30%)	402 (61.6%)	1668 (64.9%)	<.0001
National service	50 (1.23%)	2 (0.24%)	8 (1.23%)	40 (1.56%)	<.0001^a^
Personal savings	46 (1.13%)	2 (0.24%)	13 (1.99%)	31 (1.21%)	<.0001
Family/partner support	625 (15.35%)	17 (2.00%)	120 (18.4%)	488 (19.0%)	<.0001
Work	6 (0.15%)	1 (0.12%)	3 (0.4%)	2 (0.08%)	<.0001
Other	3 (0.07%)	0 (–)	1 (0.15%)	2 (0.08%)	<.0001

a Fisher’s exact calculated due to minimum cell count violations.

### Sector and Career Intentions

Table 2 displays the responses for career sectors and career intentions stratified by MD, MD-RI, and MD-PhD training status. Academia was the predominant intended career sector selected by MD-PhD trainees (85.8%). While the majority of MD-RI trainees were also interested in academic careers (57.3%), a greater proportion of MD-RIs than MD-PhDs selected private practice (17.6%) and hospitalist (12.8%) sectors. MD respondents expressed the greatest interest in private practice (36.2%) followed closely by academia (31.2%) and hospitalist (23.3%) careers. Career intentions were consistent with career sector interests. Most MD-PhD respondents indicated career intentions of basic research (22.6%), translational research (42.9%), and clinical duties (18.6%). A majority of MD-RI respondents were interested in clinical duties (51.9%), followed by clinical (14.5%) and translational research (10.7%). MD respondents were most interested in clinical duties (84.2%) followed by education (6.1%), clinical research (2.6%), and advocacy (2.6%)

**Table 2.** Career plans and intentions by MD, MD-RI, and MD-PhD

	Total, *n* (%)	MD-PhD, *n* (%)	MD-RI, *n* (%)	MD, *n* (%)	*p*-value
Sector					
Academia	1853 (47.01%)	711 (85.77%)	375 (57.34%)	767 (31.19%)	<.001
Private practice	1038 (26.33%)	32 (3.86%)	115 (17.58%)	891 (36.23%)	<.001
Consulting	75 (1.90%)	16 (1.93%)	18 (2.75%)	41 (1.67%)	<.001
Industry	68 (1.73%)	19 (2.29%)	20 (3.06%)	29 (1.18%)	<.001
Government	100 (2.54%)	12 (1.45%)	20 (3.06%)	68 (2.77%)	<.001
Hospitalist	689 (17.48%)	33 (3.98%)	84 (12.84%)	572 (23.26%)	<.001
Career intention					
Clinical duties	2564 (65.06%)	154 (18.64%)	340 (51.91%)	2070 (84.15%)	<.001
Education	227 (5.76%)	25 (3.03%)	53 (8.09%)	149 (6.06%)	<.001
Clinical research	205 (5.20%)	47 (5.69%)	95 (14.50%)	63 (2.56%)	<.001
Translational research	444 (11.27%)	354 (42.86%)	70 (10.69%)	20 (0.81%)	<.001
Basic research	206 (5.23%)	187 (22.64%)	17 (2.60%)	2 (0.08%)	<.001
Therapeutics/diagnostics	71 (1.80%)	32 (3.87%)	12 (1.83%)	27 (1.10%)	<.001
Advocacy	93 (2.36%)	5 (0.61%)	25 (3.82%)	63 (2.56%)	<.001
Administration	60 (1.52%)	3 (0.36%)	23 (3.51%)	34 (1.38%)	<.001

### Research Career Feasibility in Acute Care or Surgical Specialties

Perceptions on the feasibility of research-intense careers (defined as >70% of time dedicated to research practice) in acute care medicine and surgical specialties were compared. A greater proportion of MD-PhD and MD-RI trainees viewed balancing a research-intense career with acute care specialties (i.e., critical care, emergency medicine) as highly feasible or feasible than MD trainees (Table 3). With respect to surgical specialties, MD-PhD trainees were less likely than MD-RI and MD trainees to view balancing research-intense careers with surgical specialties as highly feasible or feasible (Table 3).

**Table 3.** Perceptions of career feasibility by MD, MD-RI, and MD-PhD

	Total, *n* (%)	MD-PhD, *n* (%)	MD-RI, *n* (%)	MD, *n* (%)	*p*-value
How feasible is a research-intense career in acute care medicine specialties (i.e. acute care, emergency medicine)?					< 0.01
Highly feasible	250 (6.33%)	65 (7.89%)	54 (8.39%)	131 (5.27%)	
Feasible	1259 (31.9%)	277 (33.6%)	221 (34.3%)	761 (30.6%)	
Difficult	1656 (41.9%)	317 (38.5%)	247 (38.4%)	1092 (44.0%)	
Highly difficult	721 (18.2%)	157 (19.1%)	110 (17.1%)	454 (18.3%)	
Impossible	66 (1.67%)	8 (0.97%)	12 (1.86%)	46 (1.85%)	
Total	3952 (100%)	824 (100%)	644 (100%)	2484 (100%)	
How feasible is a research-intense career in surgical specialties?					< 0.001
Highly feasible	256 (6.48%)	22 (2.67%)	58 (8.99%)	176 (7.09%)	
Feasible	1191 (30.1%)	116 (14.1%)	222 (34.4%)	853 (34.3%)	
Difficult	1393 (35.3%)	299 (36.3%)	198 (30.7%)	896 (36.1%)	
Highly difficult	971 (24.6%)	333 (40.5%)	148 (23.0%)	490 (19.7%)	
Impossible	141 (3.57%)	53 (6.44%)	19 (2.95%)	69 (2.78%)	
Total	3952 (100%)	823 (100%)	645(100%)	2484 (100%)	

### Specialty Intentions

MD-PhD and MD-RI trainees demonstrated similar specialty interests. MD-PhD respondents were most interested in pursuing internal medicine and related subspecialties (32.6%). This was followed by pediatrics (12.6%), neurology (10.2%), surgery and its subspecialties (9.7%), psychiatry (4.2%), and pathology (3.6%). MD-RI respondents were also most interested in pursuing internal medicine and related subspecialties (28.2%). This was followed by surgery and its subspecialties (16.4%), pediatrics (11.2%), emergency medicine (6.3%), neurology (4.57%), and psychiatry (4.3%). In contrast, MD respondents were most interested in surgery and its subspecialties (19.9%), followed by medicine and its subspecialties (19.4%), pediatrics (13.5%), emergency medicine (10.4%), family medicine (10.4%), and obstetrics and gynecology (5.5%). The remaining specialty selections for MD-PhD, MD-RI, and MD cohorts are listed in Supplementary Table 2.

### Factors Influencing Career Selection

Important factors in career selection were assessed. The top factor identified among all respondents was the ability to balance work and personal life (35.8%). The top three factors most frequently chosen among MD-PhD trainees were opportunities for research (39.8%), the ability to balance work and personal life (29.5%), and opportunities for patient care (14.7%). The top three factors for MD-RIs were opportunities to balance work and personal life (34.2%), opportunities for patient care (25.2%), and opportunities for research (12.9%). The top three factors for MDs were opportunities to balance work and personal life (39.3%), opportunities for patient care (42.5%), and financial security (3.79%). A greater proportion of MD-PhDs than MD-RIs indicated research as an important factor in choosing a career (Table 4).

**Table 4.** Career selection factor by MD, MD-RI, and MD-PhD

	Total, *n* (%)	MD-PhD, *n* (%)	MD-RI, *n* (%)	MD, *n* (%)	*p*-value
Most important factors in career selection *n* (% of 3895 total)^1^					
Ability to balance work and personal life	1407 (35.8%)	245 (29.5%)	233 (34.2%)	939 (38.3%)	<.0001
Opportunities for patient care	1329 (33.8%)	122 (14.7%)	164 (25.2%)	1043 (42.5%)	<.0001
Financial security	165 (4.19%)	29 (3.49%)	43 (6.60%)	93 (3.79%)	<.0001
Opportunities to teach	101 (2.57%)	23 (2.77%)	9 (2.91%)	59 (2.41%)	<.0001
Opportunities for research	427 (10.9%)	331 (39.8%)	84 (12.9%)	12 (0.49%)	<.0001
Opportunities for community service	124 (3.15%)	7 (0.84%)	19 (2.91%)	98 (4.00%)	<.0001
Opportunities for international work	113 (2.87%)	16 (1.93%)	32 (4.91%)	65 (2.65%)	<.0001
Autonomy	95 (2.41%)	32 (3.85%)	24 (3.68%)	39 (1.59%)	<.0001
Opportunities for student interactions	36 (0.91%)	2 (0.24%)	11 (1.69%)	23 (0.94%)	<.0001
Prestige	14 (0.36%)	0 (–)	5 (0.77%)	9 (0.37%)	<.0001^a^
Opportunities for travel	24 (0.61%)	5 (0.60%)	4 (0.61%)	15 (0.61%)	<.0001^a^
Opportunities for local work	19 (0.48%)	2 (0.24%)	5 (0.77%)	12 (0.49%)	<.0001^a^
Opportunities for national work	16 (0.41%)	3 (0.36%)	8 (1.23%)	5 (0.20%)	<.0001^a^

^1^Respondents could select up to *two* choices; will not sum to 100%.

a Fisher’s exact calculated due to minimum cell count violations.

Significant differences were also found in the perception of the importance of mentorship and ability to identify a mentor between MD-PhD, MD-RI, and MD trainees (Supplemental Table 3). MD-PhD respondents were significantly more likely to identify a mentor who helped them progress toward and/or achieve career goals (91.5%) than MD (70.6%) or MD-RI (79.4%) respondents (*p* < 0.001), and MD-PhDs (59.2%) were also more likely to say mentorship was very important in their careers thus far vs. MD-RI (49%) vs. MD (36.5%) (*p* < 0.001).

### Obstacles and Responsibilities

We then evaluated the experienced and predicted obstacles identified by MD, MD-RI, and MD-PhD cohorts. Balancing family and work responsibilities was the top experienced obstacle for all three groups (MD 35.5% vs. MD-RI 38.1% vs. MD-PhD 34.8%). While MD-PhD (28.5%) and MD-RI (27.6%) respondents were next most likely to experience challenges balancing clinical, research, and education responsibilities, MD respondents selected loan repayment (20.0%) as their second most experienced obstacle. With respect to predicted obstacles, concerns regarding balancing family and work responsibilities were again the top choice in each of the three groups (MD 55.1% vs. MD-RI 46.5% vs. MD-PhD 35.7%). While loan repayment was the second most concerning predicted obstacle for MD (17.1%) and MD-RI trainees (16.1%), lack of opportunity/funding was chosen by MD-PhD trainees (27.2%). The remaining experienced and predicted obstacles are listed in Table 5.

**Table 5.** Experienced and predicted obstacles by MD, MD-RI, and MD-PhD

	Total, *n* (%)	MD-PhD, *n* (%)	MD-RI, *n* (%)	MD, *n* (%)	*p*-value
Experienced obstacles^1^					
Balancing family and work responsibilities	1585 (35.8%)	300 (34.8%)	251 (38.1%)	1034 (35.5%)	>.05
Balance clinical, research, and education responsibilities	897 (20.2%)	246 (28.5%)	182 (27.6%)	469 (16.1%)	<.0001
Loan repayment	779 (17.6%)	48 (5.56%)	162 (24.6%)	569 (20.0%)	<.0001
Lack of opportunity/funding	720 (16.2%)	157 (18.2%)	151 (22.9%)	412 (14.2%)	<.0001
Satisfactory professional development	389 (8.78%)	103 (11.9%)	93 (14.1%)	193 (6.63%)	<.0001
Under-compensation	271 (6.11%)	82 (9.50%)	49 (7.44%)	140 (4.81%)	<.0001
Discrimination/biases (sex/ethnicity)	289 (6.52%)	74 (8.57%)	47 (7.13%)	168 (5.77%)	0.0108
Not finding position in desired location	331 (7.47%)	85 (9.85%)	57 (8.65%)	189 (6.49%)	<.01
Sexual harassment	51 (1.15%)	13 (1.51%)	14 (2.12%)	24 (0.82%)	0.0102^a^
Malpractice/lawsuit	28 (0.63%)	1 (0.12%)	4 (0.61%)	23 (0.79%)	0.0894^a^
Predicted obstacles *n* (% of 3918 total)^1^					
Balancing family and work responsibilities	1942 (49.6%)	295 (35.7%)	303 (46.5%)	1344 (55.1%)	<.0001
Balance clinical, research, and education responsibilities	351 (8.96%)	168 (20.3%)	68 (10.4%)	115 (4.71%)	<.0001
Not finding position in desired location	362 (9.24%)	65 (7.87%)	62 (9.51%)	235 (9.63%)	<.0001
Loan repayment	537 (13.7%)	16 (1.94%)	105 (16.1%)	416 (17.1%)	<.0001
Under-compensation	141 (3.60%)	10 (1.21%)	20 (3.07%)	111 (4.55%)	<.0001
Lack of opportunity/funding	333 (8.50%)	225 (27.2%)	48 (7.36%)	60 (2.46%)	<.0001
Malpractice/lawsuit	61 (1.56%)	2 (0.24%)	14 (2.15%)	45 (1.84%)	<.0001
Satisfactory professional development	101 (2.58%)	26 (3.15%)	18 (2.76%)	57 (2.34%)	<.0001
Discrimination/biases (sex/ethnicity)	47 (1.20%)	10 (1.21%)	7 (1.07%)	10 (1.23%)	<.0001
Sexual harassment	3 (0.08%)	1 (0.12%)	2 (0.31%)	0 (–)	<.0001^a^

1 Respondents could select up to *two* choices; will not sum to 100%.

a Fisher’s exact calculated due to minimum cell count violations.

Foreseeable non–work-related responsibilities during and after residency significantly differed between MD-PhD, MD-RI, and MD cohorts (Supplemental Table 4). Although raising children was viewed as a major non–work-related responsibility for all trainees, a greater proportion of MD-PhD (77.1%) respondents identified this as an important factor during residency compared with MD-RI (59.3%) and MD (54.1%) respondents. Other responsibilities including taking care of elderly parents, being a caretaker, and providing financial support are listed in Supplementary Table 4.

### Factors Associated with Career Intentions

Multivariate logistic regression analyses were performed to identify factors independently associated with academic and research career intentions. After controlling for other demographic and career factors, MD-PhD (OR 5.1, 95% CI 3.5–7.4) and MD-RI (OR 1.6, 95% CI 1.3–2.0) training status were each significantly associated with academic career intentions compared to MD status. In addition to trainee status, the ability to identify a mentor was also significantly predictive of the intention to pursue an academic career (OR 1.6, 95% CI 1.3–1.9).

MD-PhD trainees were significantly more likely than MD-RI and MD trainees to intend careers in basic science and translational research even after controlling for other demographic, career, and specialty interests by multivariate regression (Supplementary Tables 6–7). Clinical research intention was not significantly different between MD, MD-RI, and MD-PhD trainees in our models. Interestingly, while the ability to identify a mentor was associated with increased odds of translational (OR 1.4, 95% CI 1.0–1.8) and clinical research intentions (OR 1.5, 95% CI 1.2–1.9), it was not predictive of basic research career intention (OR 1.4, 95% CI 0.9–2.1) (Supplementary Tables 6–8).

### Research and Career Intention across Training Stages

Interest in academic careers was greater among respondents in later stages of medical school compared to earlier stages (MS1–2, 37.1% vs. MS3–4, 50.0%). This trend was also observed among graduate students (GS) (GS1–3, 81.7% vs. GS4+, 85.7%). There was a decline in hospitalist career interest in more senior medical students (MS1–2, 24.1% vs. MS3–4, 12.1%), and a similar decline was seen between early to late graduate students (GS1–3, 4.8% vs. GS4+, 2.5%). Interest in clinical duties remained similar across training stages. Interest in basic research increased with more senior graduate students (GS1–3, 18.7% vs. GS4+, 23.9) and dropped between MS1 (2 4%) and MS3 (4 2.2%) years. Clinical research interest was similar between MS1–2 (4.7%) and MS3–4 (4.6%). A summary of other responses, including intended research effort, segregated by training stage is included in Table 6.

**Table 6.** Research and career intention across training stages

	MS1–2, *n* (%)	GS1–3, *n* (%)	GS4+, *n* (%)	MS3–4, *n* (%)	*p*-value
Sector					
Academia	778 (37.1%)	255 (81.7%)	102 (85.7%)	643 (50.0%)	<.0001
Private practice	602 (28.7%)	12 (3.9%)	8 (6.7%)	400 (30.8%)	
Consulting	45 (2.1%)	6 (1.9%)	0 (0.0%)	23 (1.8%)	
Industry	37 (1.8%)	10 (3.2%)	1 (0.8%)	18 (1.4%)	
Government	72 (3.4%)	8 (2.6%)	3 (2.5%)	25 (1.9%)	
Hospitalist	506 (24.1%)	15 (4.8%)	3 (2.5%)	157 (12.1%)	
Career intention					
Clinical duties	1467 (70.0%)	62 (20.0%)	25 (21.4%)	938 (72.5%)	<.0001
Education	100 (4.8%)	10 (3.2%)	8 (6.8%)	104 (8.0%)	
Clinical research	99 (4.7%)	22 (7.1%)	4 (3.4%)	60 (4.6%)	
Translational research	184 (8.8%)	127 (41.0%)	42 (35.9%)	81 (6.3%)	
Basic research	83 (4.0%)	58 (18.7%)	28 (23.9%)	29 (2.2%)	
Therapeutics/diagnostics	35 (1.7%)	15 (4.8%)	5 (4.3%)	15 (1.2%)	
Advocacy	59 (2.8%)	4 (1.3%)	0 (0.0%)	25 (1.9%)	
Administration	30 (1.4%)	3 (1.0%)	1 (0.9%)	24 (1.9%)	
Career intention time allocation: research/clinical ratios					
100/0	28 (1.3%)	10 (3.1%)	6 (4.9%)	22 (1.6%)	0.0026
75/25	266 (12.0%)	144 (44.9%)	55 (44.7%)	130 (9.5%)	<.0001
50/50	324 (14.6)	106 (33.0%)	30 (24.4%)	191 (14.0%)	<.0001
25/75	1018 (45.9%)	53 (16.5%)	28 (22.8%)	656 (48.1%)	<.0001
0/100	541 (24.4%)	5 (1.6%)	2 (1.6%)	351 (25.7%)	<.0001

## Discussion

The objective of this study was to examine the factors associated with academic and research career interests in a nationally representative cohort of predoctoral physician-scientist trainees. Using a previously validated pilot survey, we evaluated current trainee career intentions and perceived obstacles to those intentions. Our results demonstrate that significant differences exist between MD trainees, MD trainees with research-intense career intentions (MD-RI), and MD-PhD trainees. Given the long-standing decline of the physician-scientist population, these data provide important insights into the perceptions of research and academic careers among trainees early in the physician-scientist career path.

### Demographics

Demographic characteristics of this study cohort are largely consistent with the expected demographics of current medical trainees in the United States. Although the proportion of women matriculating into medical school has steadily increased in recent years, female trainees remain relatively underrepresented among applicants and matriculants in MD-PhD programs. In the present study, female trainees comprised 59.7% of MD and 56.1% of MD-RI respondents but only 47.6% of MD-PhD respondents. An additional demographic consideration of this sample population is that most respondents were in the early stages of career training. First- and second-year trainees comprised 53.4% of all respondents. As expected, this bias toward earlier stages of training was driven largely by the MD (59.4%) and MD-RI (55.3%) groups, as more MD-PhD respondents were in either graduate training (47.2%) or later years of the medical school (17.8%).

### Specialty Intentions

Given the relationship between clinical specialty and the pursuit of research-oriented careers, we evaluated the specialty interests among predoctoral MD, MD-RI, and MD-PhD trainees. We found that specialty interests differed between these cohorts. While internal medicine and its subspecialties were the most frequently indicated specialties among MD-RI (28.3%) and MD-PhD (32.6%) trainees, surgery and its subspecialties were most indicated among MD-only (19.9%) trainees. Following medicine, MD-RIs were most interested in surgery and its subspecialties (16.4%), pediatrics (11.2%), emergency medicine (6.3%), and neurology (4.57%), while MD-PhD trainees were most interested in pediatrics (12.6%), neurology (10.2%), surgery and its subspecialties (9.7%), and psychiatry (4.2%). The strong interest in medicine-related specialties among research-oriented MD-RI and MD-PhD trainees is consistent with previous reports [2,16,20–22].

One notable area of difference in specialty intentions of research-oriented trainees between this study and previous studies on career outcomes of MD-PhD program graduates is in pathology. Like internal medicine, pathology has generally been regarded as a conventional specialty destination for physician-scientists. This is supported by a recent AAMC National MD-PhD Outcomes study, which identified pathology as the second most common specialty among MD-PhD program graduates (13.2%) [20–22]. By comparison, only 1.7% of MD-RI and 3.6% of MD-PhD trainees expressed an interest in this specialty in the present study. As described by others, it is plausible that this discrepancy is part of a broader trend among recent graduates away from traditional physician-scientist specialties and toward other fields such as surgery [18,25]. However, given the cross-sectional nature of this study, we are unable to conclude this. Additional longitudinal assessments aimed at understanding both the rationale underlying specialty interests and the eventual specialty destinations of predoctoral trainee cohorts are warranted.

### Research Interest

In addition to differences in intended specialties, we also found that MD, MD-RI, and MD-PhD trainees demonstrated significant differences in research interest and commitment to research careers (Supplementary Table 1). Opportunities for research was viewed as an important career selection factor for 39.8% of MD-PhD, 12.9% of MD-RI, and 0.5% of MD trainees. Conversely, opportunities for patient care demonstrated an inverse trend, with 14.7% of MD-PhD, 25.2% of MD-RI, and 42.5% of MD trainees indicating this as an important career selection factor. While MD-PhD trainees are expected to maintain a significantly greater interest in research compared with MD trainees, the relatively low level of importance placed on research opportunities among research-oriented MD-RI trainees in this study is striking and suggests that single-degree physician-scientist trainees may be less committed to research careers than their MD-PhD counterparts. Considering that single-degree physician-scientists constitute a significant proportion of the physician-scientist workforce, these data indicate that persistent efforts to expose and mentor single-degree physician-scientist trainees in areas of research may be crucial for maintaining the physician-scientist pipeline in the future.

MD-PhD and MD-RI trainees also expressed diverging interests in the types of research they intend to pursue. Overall, 65.5% of MD-PhD trainees intend careers in basic and translational research. This interest is consistent with recent data from the AAMC, which demonstrated that the majority of MD-PhD program graduates actively participate in basic and translational research efforts [20]. In comparison, only 13.3% of MD-RIs indicated an interest in basic or translational research. Instead, a higher proportion of MD-RIs selected clinical research as their preferred research activity (14.5%). Given the greater level of commitment required to conduct basic and translational research compared to clinical research activities, these data may, at least in part, explain the differences in the perceived importance of research opportunities as an important career selection factor between MD-PhD and MD-RI groups.

### The Balancing Act

A growing body of work suggests that physicians are increasingly dissatisfied with their work–life balance relative to the general US labor force [28]. Current trainees are likely aware of these trends, as medical school instructors/professors are members of this increasingly dissatisfied physician population. Previous studies have suggested that the growing emphasis on the importance of work–life balance is highlighted in specialty trends of medical trainees in the United States, as graduates are increasingly selecting specialties amenable to more favorable and controllable lifestyles [29–31]. In this analysis, MD, MD-RI, and MD-PhD trainees all indicated balancing family and work responsibilities as the primary predicted obstacle in their careers. As such, the results of this study support the concern with work–life balance in medical careers and suggest that this factor may serve as a top priority in career interest among current trainees.

Experienced and perceived family care obligations are a major consideration in the assessment of work–life balance. Previous studies have shown that these additional responsibilities are increasingly difficult to manage with the prolonged training periods required for medical careers in general and for physician-scientist careers in particular [32]. Therefore, while all trainee cohorts in this study reported concerns related to the care of children or elderly parents during and after residency training, it is unsurprising that a greater proportion of MD-PhD than MD-RI and MD respondents identified these as foreseeable responsibilities during residency. This is further supported by the demographic characteristics of respondents in this analysis, which demonstrate that predoctoral MD-PhD trainees tend to be older, in later stages of medical school training, and are more likely to have children during medical school compared with MD-RI and MD trainees (Supplementary Table 10). Finally, because the responsibility of child and elder care has traditionally fallen to women, previous reports suggested that these obstacles may disproportionately impact the development and advancement of women in academic and research-focused careers [32,33].

### Financial (In)security and Other Obstacles

Interestingly, MD-RI respondents cited financial security as an important factor in career selection more frequently than MD-PhD and MD respondents. Previous studies have shown that research-intense careers tend to provide lower financial compensation than purely clinical careers. Owing to the fact that MD-RI trainees are generally responsible for paying medical school tuition, the financial implications of pursuing less-well-compensated, research-intense careers is likely to disproportionately impact MD-RI trainees than MD-PhD trainees [34,35]. As the burden of physician debt continues to grow, MD-RI trainees may feel less secure navigating a successful research career compared to MD-PhD trainees due, in part, to a lack of sponsored formal training provided by the PhD [36]. To combat this problem, the NIH Physician-Scientist Workforce Working Group has recommended expansion of the NIH Loan Repayment Program [37]. Other options to address this concern include increased access to scholarships for MD-RI trainees and more structured research training programs to provide adequate experience for future research careers.

### Mentorship

The value of mentorship for physician-scientist trainees in this study is clear. MD-PhD trainees attribute greater importance to the mentorship they have received compared with MD-RI and MD trainees. This is possibly the result of the mentorship relationship that develops during PhD training [3,26]. In support of this notion, the ability to identify a mentor was significantly associated with academic career intentions and with interest in translational and clinical research by multivariate regression analysis. In addition to cultivating research interest, previous studies have argued that early and persistent mentorship may help address specific concerns from trainees regarding physician-scientist identity formation, as well as the challenges faced by physician-scientists within academia [38]. In this regard, a diverse and dedicated cohort of physician-scientist mentors appears to be a critical determinant of the success of efforts to cultivate a diverse next generation of physician-scientists [39].

### Limitations

A major limitation of this study is that it is a cross-sectional analysis of career intentions and interests of predoctoral trainees, which did not allow for a causative understanding of the factors that predict outcomes in this cohort. Moving forward, it will be important to do a follow-up study to see whether these differences in intentions/interests at the predoctoral level translate to differences in career outcomes and to identify what factors allow trainees to ultimately succeed in academic, research careers. Another limitation is the 27% response rate. However, this is consistent with expected response rates of social sciences surveys. Finally, several years have passed from the date of survey completion. There may have been changes in research interest, attitudes, and biomedical research policies/environment that may influence this predoctoral cohort’s career intentions. However, it is reassuring that the number of MD-PhD students and NIH funding have increased over this period, suggesting a growing interest in physician-scientist careers at the predoctoral level, and there is continued scientific support. The challenge is how to prevent attrition as physician-scientists advance in their training from predoctorates to postdoctorates, an area of active scrutiny and investigation.

## Conclusion

Significant differences in the perceptions of academic and research careers exist between predoctoral MD trainees, MD trainees interested in research careers (MD-RI), and MD-PhD trainees. Although both MD-RI and MD-PhD trainees express an interest in research, the two groups vary in the type of research they intend to pursue. A greater proportion of MD-PhD trainees intend careers in basic and translational research, while a greater proportion of MD-RI trainees intend clinical research. Outside of research interest, MD-RI and MD-PhD trainees share many of the same perspectives on training and career obstacles. However, financial concerns tend to be more prevalent among MD-RIs, and family care responsibilities during training tend to be more prevalent among MD-PhDs. It is reassuring to see increasing interest in academic career intentions from earlier training stages to later training stages. Research interest also remains stable across training stages. Overall, this study provides important insights into trainee perceptions of academic and research career pathways. These insights can be leveraged by policy-making bodies and institutions to shape policies and practices that would help retain physician-scientists.

## Supplementary material

For supplementary material accompanying this paper visit https://doi.org/10.1017/cts.2020.18. click here to view supplementary material

## References

[1] Straus SE , Straus C , Tzanetos K . Career choice in academic medicine: systematic review. Journal of General Internal Medicine 2006; 21(12): 1222–1229. doi: 10.1111/j.1525-1497.2006.00599.x 17105520 PMC1924755

[2] Andriole DA , Whelan AJ , Jeffe DB . Characteristics and career intentions of the emerging MD/PhD workforce. JAMA The Journal of the American Medical Association 2008; 300(10): 1165–1173. doi: 10.1001/jama.300.10.1165 18780845

[3] Wyngaarden JB . The clinical investigator as an endangered species. The New England Journal of Medicine 1979; 301(23): 1254–1259. doi: 10.1056/NEJM197912063012303 503128

[4] Schafer AI . The vanishing physician-scientist? Translational Research 2010; 155(1): 1–2. doi: 10.1016/j.trsl.2009.09.006 20004354 PMC2796254

[5] Milewicz DM , et al. Rescuing the physician-scientist workforce: the time for action is now. The Journal of Clinical Investigation 2015; 125(10): 3742–3747. doi: 10.1172/JCI84170 26426074 PMC4607120

[6] Jain MK , et al. Saving the endangered physician-scientist — a plan for accelerating medical breakthroughs. The New England Journal of Medicine 2019; 381(5): 399–402. doi: 10.1056/NEJMp1904482 31365796

[7] Dickler HB , et al. New physician-investigators receiving National Institutes of Health Research Project Grants: a historical perspective on the “endangered species”. JAMA The Journal of the American Medical Association 2007; 297(22): 2496–2501. doi: 10.1001/jama.297.22.2496 17565084

[8] Garrison HH , Deschamps AM . NIH research funding and early career physician scientists: continuing challenges in the 21st century. The FASEB Journal 2014; 28(3): 1049–1058. doi: 10.1096/fj.13-241687 24297696 PMC3929670

[9] Carr PL , et al. Relation of family responsibilities and gender to the productivity and career satisfaction of medical faculty. Annals of Internal Medicine 1998; 129(7): 532–538. doi: 10.7326/0003-4819-129-7-199810010-00004 9758572

[10] Shanafelt TD , et al. Burnout and satisfaction with work-life balance among US physicians relative to the general US population. Archives of Internal Medicine 2012; 172(18): 1377–1385. doi: 10.1001/archinternmed.2012.3199 22911330

[11] Bucklin BA , et al. Predictors of early faculty attrition at one Academic Medical Center. BMC Medical Education 2014; 14(1): 27. doi: 10.1186/1472-6920-14-27 24512629 PMC3923102

[12] Thorndyke LE , et al. Empowering junior faculty: Penn State’s Faculty development and mentoring program. Academic Medicine 2006; 81(7): 668–673. doi: 10.1097/01.ACM.0000232424.88922.df 16799296

[13] Johnson KS , et al. The junior faculty laboratory: an innovative model of peer mentoring. Academic Medicine 2011; 86(12): 1577–1582. doi: 10.1097/ACM.0b013e31823595e8 22030756 PMC3680343

[14] Robboy SJ , McLendon R . Structured annual faculty review program accelerates professional development and promotion: long-term experience of the Duke University Medical Center’s Pathology Department. Academic pathology 2017; 4: 2374289516689471. doi: 10.1177/2374289516689471 28725786 PMC5497916

[15] Rubio DM , et al. A comprehensive career-success model for physician-scientists. Academic Medicine 2011; 86(12): 1571–1576. doi: 10.1097/ACM.0b013e31823592fd 22030759 PMC3228877

[16] Paik JC , Howard G , Lorenz RG . Postgraduate choices of graduates from medical scientist training programs, 2004-2008. JAMA The Journal of the American Medical Association 2009; 302(12): 1271–1273. doi: 10.1001/jama.2009.1355 19773561 PMC2778489

[17] Harding CV. , Akabas MH , Andersen OS . History and outcomes of 50 years of physician-scientist training in medical scientist training programs. Academic Medicine 2017; 92(10): 1390–1398. doi: 10.1097/ACM.0000000000001779 28658019 PMC5617793

[18] Brass LF , et al. Are MD-PhD programs meeting their goals? An analysis of career choices made by graduates of 24 MD-PhD programs. Academic Medicine 2010; 85(4): 692–701. doi: 10.1097/ACM.0b013e3181d3ca17 20186033 PMC4441397

[19] Jeffe DB , Andriole DA . A national cohort study of MD-PhD graduates of medical schools with and without funding from the national institute of general medical sciences’ medical scientist training program. Academic Medicine 2011; 86(8): 953–961. doi: 10.1097/ACM.0b013e31822225c5 21694566 PMC3145809

[20] Akabas MH , Tartakovsky I , Brass LF . The national MD-PhD program outcomes study. American Association of Medical Colleges Reports [Internet] [cited Dec 9, 2019]. (https://www.aamc.org/download/489886/data/nationalmd-phdprogramoutcomesstudy.pdf)

[21] Brass LF , Akabas MH . The national MD/PhD program outcomes study: relationships between medical specialty, training duration, research effort, and career paths. JCI Insight 2019; 4(19): e133009. 10.1172/jci.insight.133009 PMC6795497 31578310

[22] Akabas MH , Brass LF . The national MD/PhD program outcomes study: outcomes variation by gender, race and ethnicity. JCI Insight 2019; 4(19): e133010. 10.1172/jci.insight.133010 PMC6795407 31578303

[23] Ahn J , et al. MD-PhD students in a major training program show strong interest in becoming surgeon-scientists. Clinical Orthopaedics and Related Research 2004; (425): 258–263. doi: 10.1097/00003086-200408000-00037 15292817

[24] Watt CD , et al. Educational views and attitudes, and career goals of MD-PhD students at the University of Pennsylvania School of Medicine. Academic Medicine 2005; 80(2): 193–198. doi: 10.1097/00001888-200502000-00019 15671328

[25] Ahn J , et al. Educating future leaders of medical research: analysis of student opinions and goals from the MD-PhD SAGE (Students’ Attitudes, Goals, and Education) survey. Academic Medicine 2007; 82(7): 633–645. doi: 10.1097/ACM.0b013e318065b907 17595558

[26] Kwan JM , et al. Exploring intentions of physician-scientist trainees: factors influencing MD and MD/PhD interest in research careers. BMC Medical Education 2017; 17(1): 115. doi: 10.1186/s12909-017-0954-8 28697782 PMC5505137

[27] National Institutes of Health. PA-16-193: NIH Pathway to Independence Award (Parent K99/R00) [Internet] [cited Jan 14, 2018]. (https://grants.nih.gov/grants/guide/pa-files/PA-16-193.html)

[28] Shanafelt TD , et al. Changes in burnout and satisfaction with work-life balance in physicians and the general US working population between 2011 and 2014. Mayo Clinic Proceedings 2015; 90(12): 1600–1613. doi: 10.1016/j.mayocp.2015.08.023 26653297

[29] Schwartz RW , et al. Controllable lifestyle: a new factor in career choice by medical students. Academic Medicine 1989; 64(10): 606–609. 2789604

[30] Dorsey ER , Jarjoura D , Rutecki GW . Influence of controllable lifestyle on recent trends in specialty choice by US medical students. JAMA The Journal of the American Medical Association 2003; 290(9): 1173–1178. doi: 10.1001/jama.290.9.1173 12952999

[31] Newton DA , Grayson MS , Thompson LF . The variable influence of lifestyle and income on medical students’ career specialty choices: data from two U.S. medical schools, 1998-2004. Academic Medicine 2005; 80(9): 809–814. doi: 10.1097/00001888-200509000-00005 16123458

[32] Colletti LM , Mulholland MW , Sonnad SS . Perceived obstacles to career success for women in academic surgery. Archives of Surgery 2000; 135(8): 972–977. doi: 10.1001/archsurg.135.8.972 10922261

[33] Bellini LM , et al. Stresses and workplace resources for academic junior faculty: track and gender comparisons. Academic Medicine 2001; 76(10 Suppl): S62–S64. doi: 10.1097/00001888-200110001-00021 11597875

[34] Gunderman RB . The perils of paying academic physicians according to the clinical revenue they generate. Medical Science Monitor 2004; 10(2): RA15–RA20. 14737057

[35] Kairouz VF , et al. Assessment of faculty productivity in academic departments of medicine in the United States: a national survey. BMC Medical Education 2014; 14(1): 205. doi: 10.1186/1472-6920-14-205 25257232 PMC4189191

[36] Association of American Medical Colleges. AAMC data book: statistical information related to medical schools and teaching hospitals [Internet], 2012 [cited Jan 14, 2018]. (https://search.library.wisc.edu/catalog/999765024602121)

[37] Gingsburg D , et al. Physician-Scientist Workforce Working Group Report. National Institutes of Health [Internet], 2014 [cited Jan 14, 2018]. (http://report.nih.gov/workforce/psw/index.aspx)

[38] Rosenblum ND , Kluijtmans M , Ten Cate O . Professional identity formation and the clinician-scientist: a paradigm for a clinical career combining two distinct disciplines. Academic Medicine 2016; 91(12): 1612–1617. doi: 10.1097/ACM.0000000000001252 27254011

[39] Andriole DA , et al. Attrition during graduate medical education: medical school perspective. Archives of Surgery 2008; 143(12): 1172–1177. doi: 10.1001/archsurg.143.12.1172 19075168

