# Supplementary material for: A nationwide assessment of perceptions of research-intense academic careers among predoctoral MD and MD-PhD trainees
Source: J Clin Transl Sci. 2020 Mar 4;4(4):307–16. doi: 10.1017/cts.2020.18 (PMC7681110; doi:10.1017/cts.2020.18)
Supplement: Supplementary file 1 [file S2059866120000187sup.zip › S2059866120000187sup001.pdf]

2012-2014 Tomorrow's Physician Survey National FINAL

Email Invitation

Dear Colleagues,

In an effort to promote the career vitality of all physicians and physician scientists, the American Physician Scientists Association (APSA) is jointly surveying current MD/DO and MD-PhD/DO-PhD program trainees to identify factors that either help or hinder their advancement in academic medicine as well as to gauge career desires of future physicians and physician-scientists. This initiative is partly in collaboration with the American Medical Women's Association (AMWA). By identifying these factors, we will be better able to *advocate for changes* that will help *reduce* the attrition of physicians in academic medicine as well as develop policy recommendations that would go toward making academic medical careers more appealing to future generations of physicians. Results will also be used to advocate for policies that will help ensure success in various specialties and career sectors.

Thank you very much for your participation.

<u>Here is some background behind this survey:</u>

• This Survey is intended to be distributed to MD/DO and MD-PhD/DO-PhD program trainees in the United States.
• The results of the survey will be used to assess the need for changes to policies/institutional structures that contribute to the attrition of physicians in academic medicine and identify factors that will help retain both men and women faculty. Results will also be used to advocate for changes to promote success in various specialties and career sectors.
• The results of the survey will be used to advocate for policies that help retain and advance a diverse academic medical faculty.
•This survey is completely anonymous and voluntary.

Collaborating Research Institutions of this Study: University of Illinois Chicago, University of Pennsylvania

When you finish the survey, you have the *option* to enter your <u>medical school email address</u>. Your email address will NOT be connected to your survey responses. Your email address will be <u>de-identified</u> from your responses.

One participant from each participating institution will be selected at random to receive and awarded a gift (i.e. $50 Amazon gift certificate).

Please follow the link below to indicate whether you consent to take the survey.

Consent

*This is a survey on behalf of the American Physician Scientists Association (APSA).
This Survey is intended to be distributed to MD, DO and MD/PhD, DO/PhD program trainees in the United States.
The results of the survey will be used to assess the need for changes to policies/institutional structures that contribute to the attrition of physicians in academic medicine and identify factors that will help retain both men and women faculty.
The results of the survey will be used to advocate for policies that help retain and advance a diverse academic medical faculty.
Results will also be used to advocate for policies that will help ensure success in various specialties and career sectors.
Any significant results will potentially be published in a peer reviewed journal.
This survey is completely anonymous and voluntary. No personally identifying information will be linked to your survey responses. There is no harm or risk associated with taking the survey. To protect the surveytaker, the account in which responses are stored is password protected and can only be accessed by the researchers.

The survey will take approximately 10 minutes to complete.

If you have any questions, please feel free to contact the following research coordinators of this study: University of Illinois, Chicago research coordinator of this study, Jennifer Kwan, jennifer.kwan@physicianscientists.org
or the University of Illinois, Chicago, Office for the Protection of Research Subjects:
203 Administrative Office Building - M/C 672
1737 West Polk Street
Chicago, IL 60612
(312) 996-1711.

University of Pennsylvania research coordinator of this study, Dania Daye, dania.daye@gmail.com, or the University of Pennsylvania, Office for the Protection of Research Subjects:
University of Pennsylvania
Office of Regulatory Affairs
Institutional Review Board
3624 Market Street, Suite 301 South
Philadelphia, PA 19104-6006

Telephone: (215) 898-2614

***Agreement of Subject**
I have read the above information and understand the terms of my participation. I agree to participate in this study.

- Yes
- No

Agreement Statement

You must have read and agreed with the statement to continue with the survey.

Thank you.

Tomorrow's Physician

Thank you for taking this 10 minute survey to identify attitudes and perceptions of trainees in medicine as well as career & life desires of future physicians.

Your participation will help us identify factors that help or hinder career advancement in medicine as well as identify what both men and women want from their future careers and allow us to take steps toward retaining diverse talents and skills and appropriate policy recommendations and changes.

Note: Please fill out this survey and the raffle page ONLY ONCE (multiple/duplicate entries will result in disqualification from entry into the raffle). Thank you very much for your participation.

Demographics

What is your age in years? Please select the relevant answer.

- <18
- 19-24
- 25-30
- 31-35
- 36+

Please select your year in Training during the 2012-2013 Academic Year.

- Medical School Year 1
- Medical School Year 2
- Medical School Year 3
- Medical School Year 4
- Graduate School Year 1
- Graduate School Year 2
- Graduate School Year 3
- Graduate School Year 4
- Graduate School Year 5
- Graduate School Year 6+
- Year out for research
- Other
- N/A

If you selected 'Other,' please specify.

*Are you enrolled in a MD/PhD program?

- Yes
- No
- DO/PhD

Other (please specify)

If so, is your MD/PhD program a MSTP?

- Yes
- No
- I don't know

Gender Identity:

- Female
- Male
- Transfemale
- Transmale
- Queer
- Genderqueer

Other (please specify)

Sexual orientation

- Straight/heterosexual
- Gay male
- Lesbian
- Bisexual
- Queer

Other (please specify)

Was a language other than English spoken in your home while you were growing up?

- Yes
- No

Ethnicity

Are you of Hispanic, Latino, or Spanish origin?

- Yes
- No

Are you:

- White
- Black or African American
- American Indian or Alaska native
- Asian Indian
- Chinese
- Filipino
- Japanese
- Korean
- Pakistani
- Vietnamese
- Native Hawaiian
- Guamanian
- Chamorro
- Native Hawaiian
- Samoan
- Multi-racial
- Other race
- Prefer not to answer

Residence

Did you live *primarily* in the United States of America during the first 18 years of your life?

- ○ Yes
- ○ No

US Zip Code

What is the US zip code of the *primary* city in which you lived during the *first 18 years* of your life?

Zip Code

Non-US

What nation/country did you primarily live in during the first 18 years of your life?

Medical School Payment

How did you primarily pay for medical school?

- ○ MD-PhD or DO-PhD program sponsored
- ○ Scholarships
- ○ Grants
- ○ Loans
- ○ National Service
- ○ Personal savings
- ○ Family/Partner support
- ○ Work
- ○ Work study
- ○ Other

Other (please specify)

What other sources of support did you have to pay for medical school? Please check all that apply.

- ☐ MD-PhD or DO-PhD program sponsored
- ☐ Scholarships
- ☐ Grants
- ☐ Loans
- ☐ National Service
- ☐ Personal savings
- ☐ Family/Partner support
- ☐ Work
- ☐ Work study
- ☐ Other
- ☐ N/A

Other (please specify)

Mother Employment

Did your mother (or female guardian) have paid employment while you were growing up?

- ○ Yes
- ○ No
- ○ N/A

Mother Employment %

During this time, did your mother (or female guardian) work <u>full-time</u> (35 hours or more per week) or <u>part-time</u> (less than 35 hours per week)?

- Full time
- Part time
- N/A

Father Employment

Did your father (or male guardian) have paid employment while you were growing up?

- Yes
- No
- N/A

Father Employment %

During this time, did your father (or male guardian) work full-time (35 hours or more per week) or part-time (less than 35 hours per week)?

- Full time
- Part time
- N/A

Mother Education

What is the *highest* education level of your mother (or female guardian)?

- Advanced degree
- College
- Some college
- High School or less
- Don't know
- N/A

Mother Advanced Degree

Please *specify* the advanced degree (s) of your mother (or female guardian).

- ☐ MD or DO
- ☐ DDS
- ☐ PhD
- ☐ JD
- ☐ DVM
- ☐ Masters
- ☐ Other
- ☐ N/A

Other (please specify)

Mother Area of Medicine

What area (s) in medicine is your mother (or female guardian) working in or did she work in?

- ☐ Consulting
- ☐ Academia
- ☐ Industry
- ☐ Government
- ☐ Private practice
- ☐ Hospitalist
- ☐ Other
- ☐ N/A

If Other (please specify)

Father Education

What is the *highest* education level of your father (or male guardian)?

- Advanced degree
- College
- Some college
- High School or less
- Don't know
- N/A

Father Advanced Degree

Please *specify* the advanced degree (s) of your father (or male guardian).

- ☐ MD or DO
- ☐ DDS
- ☐ PhD
- ☐ JD
- ☐ DVM
- ☐ Masters
- ☐ Other
- ☐ N/A

Other (please specify)

Father Area of Medicine

What area (s) in medicine is your father (or male guardian) working in or did he work in?

- ☐ Consulting
- ☐ Academia
- ☐ Industry
- ☐ Government
- ☐ Private practice
- ☐ Hospitalist
- ☐ Other
- ☐ N/A

If Other (please specify)

Are you married or partnered?

- Yes
- No

Do you have a child/children?

- Yes
- No

Children Age

What is/are the age (s) in years of your child/children?

1st Child

2nd Child

3rd Child

Personal & Professional Intentions

Do you plan to go onto residency training?

- Yes
- No

Residency Intentions

Please select your TOP residency of interest.

- Allergy and Immunology
- Anesthesiology
- Colon and Rectal Surgery
- Dermatology
- Emergency Medicine
- Family Medicine
- Internal Medicine
- Internal Medicine-Cardiology
- Internal Medicine-Endocrinology
- Internal Medicine-Gastroenterology
- Internal Medicine-Hematology/Oncology
- Internal Medicine-Infectious Disease
- Internal Medicine-Pulmonology
- Internal Medicine-Rheumatology
- Medical Genetics
- Neurological Surgery
- Neurology
- Nuclear Medicine
- Obstetrics and Gynecology
- Ophthalmology
- Orthopaedic Surgery
- Otolaryngology
- Pathology
- Pediatrics
- Physical Medicine and Rehabilitation
- Plastic Surgery
- Preventive Medicine
- Psychiatry
- Radiation Oncology

- Radiology
- Surgery
- Thoracic Surgery
- Urology
- Other

Other (please specify)

Please select your SECOND residency of interest.

- Allergy and Immunology
- Anesthesiology
- Colon and Rectal Surgery
- Dermatology
- Emergency Medicine
- Family Medicine
- Internal Medicine
- Internal Medicine-Cardiology
- Internal Medicine-Endocrinology
- Internal Medicine-Gastroenterology
- Internal Medicine-Hematology/Oncology
- Internal Medicine-Infectious Disease
- Internal Medicine-Pulmonology
- Internal Medicine-Rheumatology
- Medical Genetics
- Neurological Surgery
- Neurology
- Nuclear Medicine
- Obstetrics and Gynecology
- Ophthalmology
- Orthopaedic Surgery
- Otolaryngology
- Pathology

- Pediatrics
- Physical Medicine and Rehabilitation
- Plastic Surgery
- Preventive Medicine
- Psychiatry
- Radiation Oncology
- Radiology
- Surgery
- Thoracic Surgery
- Urology
- Other

Other (please specify)

Specialty Choice and Research Feasibility

In your opinion, how feasible do you think it is to have a *research intense career* (>70% of time dedicated to research pursuits) in *acute care medicine specialties* (i.e. critical care, Emergency Medicine)?

- Highly Feasible
- Feasible
- Difficult
- Highly Difficult
- Impossible

In your opinion, how feasible do you think it is to have a *research intense career* (>70% of time dedicated to research pursuits) in *surgical specialties* (i.e. orthopedic surgery, urology, neurosurgery)?

- Highly Feasible
- Feasible
- Difficult
- Highly Difficult
- Impossible

Post Residency Intentions

What are other foreseeable non work-related responsibilities for you DURING residency? ***Check all that apply:***

- ☐ Raising children
- ☐ Taking care of elderly parents
- ☐ Being caretaker to others
- ☐ Offering Financial support to others
- ☐ Other (please specify)

What are foreseeable non work-related responsibilities for you AFTER residency? ***Check all that apply:***

- ☐ Raising children
- ☐ Taking care of elderly parents
- ☐ Being caretaker to others
- ☐ Offering Financial support to others
- ☐ Other

(please specify)

Career Intentions

What career sector do you foresee yourself in post medical training? ***Please check the MOST likely/Highest priority one.***

- Consulting
- Academia
- Industry
- Government
- Private practice
- Hospitalist
- Other
- N/A

Other (please specify)

What career sector do you foresee yourself in post medical training? ***Please check the SECOND most likely/Second priority one.***

- Consulting
- Academia
- Industry
- Government
- Private practice
- Hospitalist
- Other
- N/A

Other (please specify)

What are your career intentions? ***Please check the MOST likely/Highest priority one.***

- Education
- Basic research
- Clinical research
- Translational research
- Clinical duties
- Therapeutics/diagnostics development
- Advocacy
- Administration
- N/A
- Other

Other (please specify)

What are your career intentions? ***Please check the SECOND most likely/Second priority one.***

- Education
- Basic research
- Clinical research
- Translational research
- Clinical duties
- Therapeutics/diagnostics development
- Advocacy
- Administration
- N/A
- Other

Other (please specify)

If you had your dream career, how would you allocate your time between research and clinical work?

	Research/Clinical Work
100/0	○
75/25	○
50/50	○
25/75	○
0/100	○

After you complete your training how many hours do you want to work/week?

- ○ Full time >35 hours
- ○ Part time <35 Hours
- ○ Not working for pay
- ○ N/A
- ○ Other

Other (please specify)

If you are (or intend to be) in a committed relationship or have a spouse, how much do you foresee your spouse/partner working?

- ○ Full time >35 hours
- ○ Part time <35 Hours
- ○ Not working for pay
- ○ N/A
- ○ Other

Other (please specify)

Career Priorities

What is the MOST important factor to you in selecting a career?

- Opportunities to do research
- Opportunities for patient care
- Opportunities to teach
- Opportunities for community service
- Opportunities for interactions with students
- Opportunities for travel
- Opportunities for international work
- Opportunities for national work
- Opportunities for local work
- Ability to balance work and personal life
- Financial security
- Autonomy
- Prestige
- Other

Other (please specify)

What is the SECOND most important factor to you in selecting a career?

- Opportunities to do research
- Opportunities for patient care
- Opportunities to teach
- Opportunities for community service
- Opportunities for interactions with students
- Opportunities for travel
- Opportunities for international work
- Opportunities for national work
- Opportunities for local work
- Ability to balance work and personal life
- Financial security
- Autonomy
- Prestige
- Other

Other (please specify)

Career Obstacles

What do you foresee as the MOST pressing obstacle in your career?

- Lack of opportunity/funding
- Not finding position in desired location
- Loan repayment
- Malpractice/lawsuit
- Under-compensation
- Discrimination/biases against your gender/ethnicity/sexual orientation
- Sexual harassment
- Balancing family and work responsibilities
- Balancing clinical, research, and education responsibilities
- Satisfactory professional advancement
- Other

Other (please specify)

What do you foresee as the SECOND MOST pressing obstacle in your career?

- Lack of opportunity/funding
- Not finding position in desired location
- Loan repayment
- Malpractice/lawsuit
- Under-compensation
- Discrimination/biases against your gender/ethnicity/sexual orientation
- Sexual harassment
- Balancing family and work responsibilities
- Balancing clinical, research, and education responsibilities
- Satisfactory professional advancement
- Other

Other (please specify)

So far in your training, what have you encountered as a hindrance to your career advancement? ***Please check all that apply:***

- [ ] Lack of opportunity/funding
- [ ] Not finding position in desired location
- [ ] Loan repayment
- [ ] Malpractice/lawsuit
- [ ] Under-compensation
- [ ] Discrimination/biases against your gender/ethnicity
- [ ] Sexual harassment
- [ ] Balancing family and work responsibilities
- [ ] Balancing clinical, research, and education responsibilities
- [ ] Satisfactory professional advancement
- [ ] Other

Other (please specify)

Views & Perceptions

In your opinion, how much importance is given to talents/accomplishments when recruiting applicants for jobs and/or positions in science and medicine?

- A great deal of importance
- A lot of importance
- Moderate amount of importance
- Little importance
- None at all

In your opinion, how much importance is given to connections/networking when recruiting applicants for jobs and/or positions in science and medicine?

- A great deal of importance
- A lot of importance
- Moderate amount of importance
- Little importance
- None at all

Mentorship

Can you currently identify a mentor (s) who has helped you progress toward &/or achieve your career goals?

- Yes
- No

To your knowledge, has your mentor (s) gone beyond giving feedback and advice and use his or her influence to advocate for you in regards to awards, fellowships, promotions?

- Yes
- No
- Maybe
- I don't know

Other (please specify)

Mentorship and Career

What gender is/are your mentor (s)

- Female
- Male
- BOTH

What position (s) do your mentors hold?

- Dean
- Department head
- Professor
- Associate Professor
- Assistant Professor
- Lecturer
- Attending
- Fellow
- Resident

Other (please specify)

If you are an underrepresented minority (URM), do you have a mentor who is also an URM?

- Yes
- No

How important has mentorship been in your training thus far?

- Very important
- Somewhat Important
- Not very important
- Not at all important

Do you have at least one mentor for *training/professional/career issues*?

- Yes
- No

Do you have at least one mentor for *work-life issues*?

- Yes
- No

Do you WANT a mentor for *work-life issues*?

- Yes
- No

Do you have at least one role model for the kind of *career and life you want*?

- Yes
- No

Views and Perceptions

We will now be asking questions pertaining to your views and perceptions of academic medicine.

Views & Perceptions

In the United States, the number of women entering academia has been steadily increasing over the past 40 years.

- True
- False
- I Don't Know

Views & Perceptions

Please select the statement that you think is *true*.

- ○ Women want DIFFERENT things from an academic career than men
- ○ Women want the SAME things from an academic career as men.

Views & Perceptions

Please select the statement that you think is *true*.

- ○ Men and women with similar accomplishments are NOT advanced/promoted at the same rate in academia.
- ○ Men and women with similar accomplishments ARE advanced/promoted at the same rate in academia.

Views & Perceptions

Please select the statement that you think is *true*.

- ◯ Women ARE equally represented as men in higher faculty positions.
- ◯ Women are NOT equally represented as men in higher faculty positions.

Views & Perceptions

Please select the statement that you think is *true*.

- Women and men ARE equally represented in leadership positions in medical institutions, associations & medical journals.
- Women and men are NOT equally represented in leadership positions in medical institutions, associations & medical journals.

Views & Perceptions

Internalized/social perceptions *influence* advancement in science and medicine.

- Strongly Agree
- Agree
- Disagree
- Strongly Disagree

Views & Perceptions

In your opinion, in the quest for an academic career, do women have access to MORE, the SAME, or FEWER resources* as do men?

Resources include: Space, support personnel, start-up funds, equipment, services.

- MORE
- SAME
- FEWER

Views & Perceptions

***In your opinion*, do you agree or disagree with the following statement? BOTH women and men desire increased flexibility and greater work-life balance in academic careers.**

- Strongly Agree
- Agree
- Disagree
- Strongly Disagree

Views & Perceptions

Please select the statement that you think is *true*.

- ○ Stereotypes do NOT contribute to the gender imbalance in leadership positions in medical school faculty.
- ○ Stereotypes DO contribute to the gender imbalance in higher positions in medical school faculty.

Views & Perceptions

In your opinion, are men compensated MORE, SAME or LESS as women for similar work?

- MORE
- SAME
- LESS

Views & Perceptions

What factors do you believe contribute to the low proportions of women full professors (18%), department chairs (13%), and medical school deans (12%)? ***Check all that you believe apply.***

- ☐ Pipeline (not enough women entering the profession of medicine)
- ☐ Lack of mentors
- ☐ Lack of sponsors (mentors who go beyond feedback & help with advocating and getting promotions)
- ☐ Lack of flexibility to balance professional/personal responsibilities
- ☐ Unwelcoming environment
- ☐ Too much service/training responsibilities
- ☐ Prefer service/training responsibilities
- ☐ Underproductive compared to men
- ☐ Women are not interested in leadership
- ☐ Ineffective negotiation or lack of negotiation.
- ☐ Sexual harassment-direct or indirect
- ☐ I Don't Know

Other (please specify)

Check all that you think apply. **Fewer women than men are hired and advanced in tenure track positions due to the following:**

- ☐ Gender stereotypes (aka schemas)
- ☐ Lack of mentors
- ☐ Lack of sponsors (mentors who go beyond feedback & help with advocating and getting promotions)
- ☐ Evaluation bias against women
- ☐ Not enough women entering the field
- ☐ Accumulation of disadvantage
- ☐ I Don't Know

Other (please specify)

Survey Comments

Please let us know if you have any feedback, comments or suggestions on the survey instrument below.

Tomorrow's Physician: Educational Highlights

We would like to share with you some of the background information and research on which the latter part of the survey was based. You will have the option to enter your email address (that may be chosen at random for a $50 amazon.com gift certificate) after the educational highlights section.
