# Supplementary material for: A nationwide assessment of perceptions of research-intense academic careers among predoctoral MD and MD-PhD trainees
Source: J Clin Transl Sci. 2020 Mar 4;4(4):307–16. doi: 10.1017/cts.2020.18 (PMC7681110; doi:10.1017/cts.2020.18)
Supplement: Supplementary file 1 [file S2059866120000187sup.zip › S2059866120000187sup002.docx]

| Supplemental Table 1 | | |  | |  | | |  |  |  |
| --- | --- | --- | --- | --- | --- | --- | --- | --- | --- | --- |
|  |  | | **Total, n (%)** | | **MD-PhD, n (%)** | | | **MD-RI, n (%)** | **MD, n (%)** | **P Value** |
| Career Intention Time Allocation: Research/Clinical Ratios n (% of 4,433 Total) | | | | | | | |  |  |  |
|  | 100/0 | | 67 (1.51%) | | 25 (2.90%) | | | 42 (6.3%) | 0 (--) | <.0001^a^ |
|  | 75/25 | | 613 (13.8%) | | 392 (46.1%) | | | 221 (33.1%) | 0 (--) | <.0001 |
|  | 50/50 | | 687 (15.5%) | | 283 (33.3%) | | | 404 (60.6%) | 0 (--) | <.0001 |
|  | 25/75 | | 1813 (40.9%) | | 128 (14.9%) | | | 0 (--) | 1686 (65.5%) | <.0001 |
|  | 0/100 | | 911 (20.6%) | | 23 (2.7%) | | | 0 (--) | 888 (34.5%) | <.0001 |
| ^1^ = Due to missing responses, will not sum to 100% | | | | |  | | |  |  |  |
|  | |  | |  | |  |  |  |  |  |

| Supplemental Table 2 | |  |  |  |  |  |
| --- | --- | --- | --- | --- | --- | --- |
|  |  | **Total, n (%)** | **MD-PhD, n (%)** | **MD-RI, n (%)** | **MD, n (%)** | **P Value** |
| Specialty^1^ | |  |  |  |  |  |
|  | Allergy and Immunology | 16 (0.41%) | 13 (1.60%) | 2 (0.32%) | 1 (0.049%) | <.0001^a^ |
|  | Anesthesiology | 153 (3.89%) | 13 (1.60%) | 28 (4.42%) | 112 (4.51%) | <.0001 |
|  | Colon and Rectal Surgery | 1 (0.03%) | 0 (--) | 1 (0.16%) | 0 (--) | <.0001^a^ |
|  | Dermatology | 96 (2.44%) | 16 (1.96%) | 11 (1.74%) | 69 (2.78%) | <.0001 |
|  | Emergency Medicine | 324 (8.24%) | 25 (3.07%) | 40 (6.31%) | 259 (10.4%) | <.0001 |
|  | Family Medicine | 297 (7.56%) | 11 (1.35%) | 29 (4.57%) | 257 (10.4%) | <.0001 |
|  | Internal Medicine | 356 (9.06%) | 55 (6.57%) | 69 (10.9%) | 232 (9.35%) | <.0001 |
|  | Internal Medicine – Cardiology | 142 (3.61%) | 30 (3.68%) | 34 (5.36%) | 78 (3.14%) | <.0001 |
|  | Internal Medicine – Endocrinology | 32 (0.81%) | 11 (1.35%) | 7 (1.10%) | 14 (0.56%) | <.0001 |
|  | Internal Medicine – Gastroenterology | 40 (1.02%) | 13 (1.60%) | 6 (0.95%) | 48 (2.12%) | <.0001 |
|  | Internal Medicine —Hematology/Oncology | 186 (4.73%) | 92 (11.3%) | 40 (6.31%) | 54 (2.18%) | <.0001 |
|  | Internal Medicine – Infectious Disease | 105 (2.67%) | 53 (6.50%) | 19 (3.00%) | 33 (1.33%) | <.0001 |
|  | Internal Medicine – Pulmonology | 19 (0.48%) | 8 (0.98%) | 3 (0.47%) | 8 (0.32%) | <.0001 |
|  | Internal Medicine – Rheumatology | 8 (0.20%) | 5 (0.61%) | 1 (0.16%) | 8 (0.35%) | <.0001 |
|  | Medical Genetics | 20 (0.51%) | 10 (1.23%) | 0 (--) | 10 (0.4%) | <.0001 |
|  | Neurological Surgery | 84 (2.14%) | 23 (2.82%) | 17 (2.68%) | 44 (1.77%) | <.0001^a^ |
|  | Neurology | 174 (4.43%) | 83 (10.2%) | 29 (4.57%) | 62 (2.5%) | <.0001 |
|  | Nuclear Medicine | 3 (0.08%) | 3 (0.37%) | 0 (--) | 0 (--) | <.0001^a^ |
|  | Obstetrics and Gynecology | 183 (4.66%) | 23 (2.82%) | 24 (3.79%) | 136 (5.48%) | <.0001 |
|  | Ophthalmology | 118 (3.00%) | 27 (3.31%) | 21 (3.31%) | 70 (2.82%) | <.0001 |
|  | Orthopedic Surgery | 151 (3.84%) | 8 (0.98%) | 20 (3.15%) | 123 (4.96%) | <.0001 |
|  | Otolaryngology | 82 (2.09%) | 13 (1.60%) | 14 (2.21%) | 55 (2.22%) | <.0001 |
|  | Pathology | 56 (1.42%) | 29 (3.56%) | 11 (1.74%) | 16 (0.64%) | <.0001 |
|  | Pediatrics | 508 (12.93%) | 103 (12.64%) | 71 (11.2%) | 334 (13.5%) | <.0001 |
|  | Physical Medicine and Rehabilitation | 36 (0.92%) | 4 (0.49%) | 9 (1.42%) | 23 (0.93%) | <.0001^a^ |
|  | Plastic Surgery | 40 (1.02%) | 3 (0.37%) | 6 (0.95%) | 31 (1.25%) | <.0001 |
|  | Preventative Medicine | 12 (0.31%) | 3 (0.37%) | 3 (0.47%) | 6 (0.24%) | <.0001^a^ |
|  | Psychiatry | 115 (2.93%) | 34 (4.17%) | 27 (4.26%) | 54 (2.18%) | <.0001 |
|  | Radiation Oncology | 56 (1.42%) | 27 (3.31%) | 11 (1.74%) | 18 (0.73%) | <.0001 |
|  | Radiology | 109 (2.77%) | 28 (3.44%) | 23 (3.63%) | 58 (2.34%) | <.0001 |
|  | Surgery | 246 (6.26%) | 24 (2.94%) | 36 (5.68%) | 186 (7.50%) | <.0001 |
|  | Thoracic Surgery | 32 (0.81%) | 2 (0.25%) | 7 (1.10%) | 23 (0.93%) | <.0001^a^ |
|  | Urology | 41 (1.04%) | 6 (0.74%) | 3 (0.47%) | 32 (1.29%) | <.0001 |
|  | Other | 89 (2.26%) | 17 (2.09%) | 12 (1.89%) | 60 (2.42%) | <.0001 |
| ^a^ = Fisher's Exact calculated due to minimum cell count violations | | |  |  |  |  |
|  | | |  |  |  |  |

| Supplemental Table 3 | | |  | |  | | |  |  |  |
| --- | --- | --- | --- | --- | --- | --- | --- | --- | --- | --- |
|  |  | | **Total, n (%)** | | **MD-PhD, n (%)** | | | **MD-RI, n (%)** | **MD, n (%)** | **P Value** |
| Can you currently identify a mentor(s) who has helped you progress toward &/or achieve your career goals? | | | | | | | | |  | < 0.001 |
|  | Yes | | 2971 (76.5%) | | 753 (91.5%) | | | 511 (79.4%) | 1707 (70.6%) |  |
|  | No | | 914 (23.5%) | | 70 (8.51%) | | | 133 (20.6%) | 711 (29.4%) |  |
|  | TOTAL | | 3885 (100%) | | 823 (100%) | | | 644 (100%) | 2418 (10%) |  |
| How important has mentorship been in your training thus far? | | | | |  | | |  |  | <.0001^a^ |
|  | Very important | | 1307 (44.4%) | | 442 (59.2%) | | | 247 (49.0%) | 618 (36.5%) |  |
|  | Somewhat important | | 1305 (44.3%) | | 263 (35.2%) | | | 209 (41.5%) | 833 (49.1%) |  |
|  | Not very important | | 308 (10.5%) | | 39 (5.22%) | | | 44 (8.73%) | 225 (13.3%) |  |
|  | Not at all important | | 26 (0.88%) | | 3 (0.40%) | | | 4 (0.79%) | 19 (1.12%) |  |
|  | TOTAL | | 2946 (100%) | | 747 (100%) | | | 504 (100%) | 1695 (100%) |  |
| How much importance is given to talents/accomplishments when recruiting applicants for jobs and/or positions in science and medicine? | | | | | | | | | | <.0001 |
|  | A great deal of importance | | 1194 (30.75%) | | 326 (39.4%) | | | 200 (31.1%) | 668 (27.7%) |  |
|  | A lot of importance | | 1876 (48.3%) | | 376 (45.5%) | | | 297 (46.2%) | 1203 (49.9%) |  |
|  | Moderate amount of importance | | 747 (19.2%) | | 114 (13.8%) | | | 130 (20.2%) | 503 (20.9%) |  |
|  | Little importance | | 3 (1.62%) | | 9 (1.09%) | | | 15 (2.33%) | 39 (1.62%) |  |
|  | None at all | | 3 (0.08%) | | 2 (0.24%) | | | 1 (0.16%) | 0 (--) |  |
|  | TOTAL | | 3883 (100%) | | 827 (100%) | | | 643 (100%) | 2413 (100%) |  |
| How much importance is given to connections/networking when recruiting applicants for jobs and/or positions in science and medicine? | | | | | | | | | | <.0001 |
|  | A great deal of importance | | 1262 (32.5%) | | 284 (34.4%) | | | 225 (34.9%) | 753 (31.2%) |  |
|  | A lot of importance | | 1638 (42.1%) | | 355 (42.9%) | | | 263 (40.8%) | 1020 (42.2%) |  |
|  | Moderate amount of importance | | 865 (22.3%) | | 177 (21.4%) | | | 132 (20.5%) | 556 (23.0%) |  |
|  | Little importance | | 121 (3.11%) | | 10 (1.21%) | | | 25 (3.88%) | 86 (3.56%) |  |
|  | None at all | | 2 (0.05%) | | 1 (0.12%) | | | 0 (--) | 1 (0.04) |  |
|  | TOTAL | | 3888 (100%) | | 827 (100%) | | | 645 (100%) | 2416 (100%) |  |
|  | |  | |  | |  |  |  |  |  |
|  | |  | |  | |  |  |  |  |  |

| Supplemental Table 4 | |  |  | |  | |  | |  |
| --- | --- | --- | --- | --- | --- | --- | --- | --- | --- |
|  |  | **Total, n (%)** | **MD-PhD, n (%)** | | **MD-RI, n (%)** | | **MD, n (%)** | | **P Value** |
| Foreseeable non-work-related responsibilities DURING residency^1^ n (% of 4,433 Total) | | | |  | |  | |  |  |
|  | Raising children | 2630 (59.3%) | 665 (77.1%) | | 391 (59.3%) | | 1574 (54.1%) | | <.0001 |
|  | Taking care of elderly parents | 769 (18.0%) | 213 (24.7%) | | 153 (23.2%) | | 430 (14.8%) | | <.0001 |
|  | Being caretaker to others | 584 (13.2%) | 102 (11.8%) | | 105 (15.9%) | | 377 (12.9%) | | 0.0524 |
|  | Financial support of others | 1141 (25.7%) | 227 (26.30%) | | 193 (29.3%) | | 721 (24.8%) | | 0.0518 |
| Foreseeable non-work-related responsibilities AFTER residency^1^ | |  |  | |  | |  | |  |
|  | Raising children | 3657 (82.5%) | 770 (89.2%) | | 591 (89.7%) | | 2296 (78.9%) | | <.0001 |
|  | Taking care of elderly parents | 2960 (60.7%) | 588 (68.1%) | | 461 (70.0%) | | 1641 (56.4%) | | <.0001 |
|  | Being caretaker to others | 1268 (28.6%) | 221 (25.6%) | | 207 (31.4%) | | 840 (28.9%) | | <.05 |
|  | Financial support of others | 2218 (50.0%) | 412 (47.7%) | | 385 (58.4%) | | 1421 (48.8%) | | <.0001 |
| ^1^ = Respondents could select all applicable choices, will not sum to 100% | |  |  | |  | |  | |  |
| ^a^ = Fisher's Exact calculated due to minimum cell count violations | |  |  | |  | |  | |  |

| Supplemental Table 5 | | |  |  |
| --- | --- | --- | --- | --- |
|  |  | | **Effects of various factors on academic career intentions** | |
| **Factor** | | | **Odds Ratio** | **95% Confidence Interval** |
|  | **MD-RI vs MD-PhD** | | **0.319** | **0.211 - 0.484** |
|  | **MD-RI vs MD** | | **1.621** | **1.27 - 2.069** |
|  | **MD-PhD vs MD** | | **5.077** | **3.47 - 7.428** |
|  | Female | | 0.847 | 0.709 - 1.013 |
|  | Hispanic | | 1.087 | 0.741 - 1.596 |
|  | **Asian or Pacific Islander vs Black or African American** | | **1.693** | **1.031 - 2.781** |
|  | Asian or Pacific Islander vs Black or Multiracial or Other | | 1.1 | 0.775 - 1.563 |
|  | Asian or Pacific Islander vs White | | 0.84 | 0.628 - 1.123 |
|  | **Black or African American vs White** | | **0.496** | **0.322 - 0.764** |
|  | Multiracial or Other vs White | | 0.763 | 0.594 - 0.98 |
|  | Paid primarily through loans | | 0.871 | 0.726 - 1.044 |
|  | **Sector: Private Practice** | | **0.203** | **0.166 - 0.248** |
|  | **Intention: Education** | | **4.471** | **3.674 - 5.439** |
|  | **Intend: Clinical Research** | | **3.766** | **2.996 - 4.732** |
|  | **Intend: Translational Research** | | **3.002** | **2.165 - 4.162** |
|  | **Intend: Basic Research** | | **4.591** | **2.717 - 7.759** |
|  | Spec: Internal Medicine | | 0.883 | 0.73 - 1.068 |
|  | Spec: Surgery | | 1.07 | 0.858 - 1.336 |
|  | Spec: Radiology | | 1.022 | 0.687 - 1.52 |
|  | **Spec: Emergency Med.** | | **0.616** | **0.487 - 0.78** |
|  | **Spec: Family Medicine** | | **0.505** | **0.394 - 0.646** |
|  | Predict Eldercare after residency | | 1.13 | 0.944 - 1.352 |
|  | **Balance Clinical, Research, Education.** | | **1.599** | **1.291 - 1.981** |
|  | Career Factor: Community Svc | | 0.77 | 0.59 - 1.003 |
|  | **Can Identify Mentor** | | **1.627** | **1.347 - 1.964** |
|  | |  |  |  |
|  | |  |  |  |

| Supplemental Table 6 | | |  |  |
| --- | --- | --- | --- | --- |
|  |  | | **Effects of various factors on basic science research aspiration** | |
| **Factor** | | | **Odds Ratio** | **95% Confidence Interval** |
|  | **MD-RI vs MD-PhD** | | **0.238** | **0.153 - 0.37** |
|  | **MD-RI vs MD** | | **7.757** | **4.716 - 12.759** |
|  | **MD-PhD vs MD** | | **32.592** | **19.568 - 54.282** |
|  | **Female** | | **0.641** | **0.483 - 0.851** |
|  | **Hispanic** | | **3.229** | **1.123 - 9.281** |
|  | **Asian or Pacific Islander vs Black or African American** | | **1.475** | **0.873 - 2.492** |
|  | Asian or Pacific Islander vs Black or Multiracial or Other | | 1.013 | 0.673 - 1.527 |
|  | Asian or Pacific Islander vs White | | 0.457 | 0.158 - 1.317 |
|  | **Black or African American vs Multiracial or Other** | | **0.314** | **0.115 - 0.857** |
|  | Black or African American vs White | | 0.687 | 0.457 - 1.034 |
|  | Multiracial or Other vs White | | 1.289 | 0.688 - 2.414 |
|  | Paid primarily through loans | | 0.699 | 0.463 - 1.057 |
|  | **Sector: Private Practice** | | **0.549** | **0.397 - 0.76** |
|  | **Intention: Education** | | **0.142** | **0.091 - 0.224** |
|  | **Intend: Clinical Research** | | **0.067** | **0.04 - 0.114** |
|  | **Intend: Translational Research** | | **0.243** | **0.171 - 0.346** |
|  | Spec: Internal Medicine | | 0.899 | 0.672 - 1.204 |
|  | **Spec: Surgery** | | **0.655** | **0.437 - 0.983** |
|  | Spec: Radiology | | 0.759 | 0.406 - 1.419 |
|  | **Spec: Emergency Med.** | | **0.218** | **0.115 - 0.41** |
|  | **Spec: Family Medicine** | | **0.201** | **0.087 - 0.464** |
|  | Predict Eldercare after res | | 1.05 | 0.773 - 1.428 |
|  | Obs: Balance Clin Res. Ed. | | 1.145 | 0.844 - 1.552 |
|  | **Career Factor: Community Svc** | | **0.26** | **0.1 - 0.676** |
|  | Can Identify Mentor | | 1.385 | 0.902 - 2.128 |
|  | |  |  |  |
|  | |  |  |  |

| Supplemental Table 7 | | |  | |  |
| --- | --- | --- | --- | --- | --- |
|  |  | | **Effects of various factors on a translational science research aspiration** | | |
| **Factor** |  | | **Odds Ratio** | | **95% Confidence Interval** |
|  | **MD-RI vs MD-PhD** | | **0.226** | | **0.166 - 0.309** |
|  | **MD-RI vs MD** | | **4.325** | | **3.198 - 5.851** |
|  | **MD-PhD vs MD** | | **19.106** | | **13.902 - 26.258** |
|  | **Female** | | **0.708** | | **0.568 - 0.884** |
|  | Hispanic | | 1.113 | | 0.692 - 1.79 |
|  | Asian or Pacific Islander vs Black or African American | | 0.922 | | 0.518 - 1.642 |
|  | Asian or Pacific Islander vs Black or Multiracial or Other | | 1.372 | | 0.909 - 2.07 |
|  | **Asian or Pacific Islander vs White** | | **1.546** | | **1.107 - 2.159** |
|  | Black or African American vs Multiracial or Other | | 1.488 | | 0.844 - 2.624 |
|  | **Black or African American vs White** | | **1.677** | | **1.004 - 2.799** |
|  | Multiracial or Other vs White | | 1.126 | | 0.825 - 1.539 |
|  | Paid primarily through loans | | 0.947 | | 0.722 - 1.241 |
|  | **Sector: Private Practice** | | **0.613** | | **0.484 - 0.778** |
|  | **Intention: Education** | | **0.159** | | **0.116 - 0.219** |
|  | **Intend: Clinical Research** | | **0.32** | | **0.245 - 0.418** |
|  | Spec: Internal Medicine | | 1.123 | | 0.886 - 1.422 |
|  | **Spec: Surgery** | | **1.628** | | **1.225 - 2.163** |
|  | Spec: Radiology | | 1.141 | | 0.714 - 1.826 |
|  | **Spec: Emergency Med.** | | **0.591** | | **0.408 - 0.855** |
|  | **Spec: Family Medicine** | | **0.31** | | **0.186 - 0.519** |
|  | Predict Eldercare after res | | 0.874 | | 0.69 - 1.106 |
|  | Obs: Balance Clin Res. Ed. | | 1.125 | | 0.882 - 1.434 |
|  | Career Factor: Community Svc | | 0.775 | | 0.496 - 1.212 |
|  | **Can Identify Mentor** | | **1.36** | | **1.006 - 1.839** |
|  | |  | |  |  |
|  | |  | |  |  |

| Supplemental Table 8 | | |  | |  |
| --- | --- | --- | --- | --- | --- |
|  |  | | **Effects of various factors on clinical research aspiration** | | |
| **Factor** |  | | **Odds Ratio** | | **95% Confidence Interval** |
|  | MD-RI vs MD-PhD | | 0.395 | | 0.292 - 0.536 |
|  | MD-RI vs MD | | 0.408 | | 0.327 - 0.51 |
|  | MD-PhD vs MD | | 0.968 | | 0.722 - 1.298 |
|  | Female | | 1.189 | | 0.995 - 1.42 |
|  | Hispanic | | 1.292 | | 0.77 - 2.165 |
|  | Asian or Pacific Islander vs Black or African American | | 1.057 | | 0.75 - 1.489 |
|  | Asian or Pacific Islander vs Black or Multiracial or Other | | 0.991 | | 0.749 - 1.312 |
|  | Asian or Pacific Islander vs White | | 0.818 | | 0.495 - 1.352 |
|  | Black or African American vs Multiracial or Other | | 0.768 | | 0.485 - 1.215 |
|  | Black or African American vs White | | 0.938 | | 0.731 - 1.203 |
|  | Multiracial or Other vs White | | 1.027 | | 0.704 - 1.499 |
|  | Paid primarily through loans | | 1.17 | | 0.966 - 1.418 |
|  | Sector: Private Practice | | 0.944 | | 0.781 - 1.14 |
|  | Intention: Education | | 0.134 | | 0.106 - 0.171 |
|  | Intend: Translational Research | | 0.291 | | 0.223 - 0.379 |
|  | Spec: Internal Medicine | | 1.166 | | 0.965 - 1.41 |
|  | **Spec: Surgery** | | **1.522** | | **1.228 - 1.886** |
|  | Spec: Radiology | | 0.95 | | 0.641 - 1.406 |
|  | **Spec: Emergency Med.** | | **0.756** | | **0.582 - 0.981** |
|  | **Spec: Family Medicine** | | **0.367** | | **0.268 - 0.503** |
|  | Predict Eldercare after res | | 1.025 | | 0.853 - 1.232 |
|  | **Obs: Balance Clin Res. Ed.** | | **1.385** | | **1.143 - 1.679** |
|  | **Career Factor: Community Svc** | | **0.484** | | **0.349 - 0.671** |
|  | **Can Identify Mentor** | | **1.499** | | **1.214 - 1.851** |
|  | |  | |  |  |
|  | |  | |  |  |

| Supplemental Table 9 | | |  | |  |
| --- | --- | --- | --- | --- | --- |
|  |  | | **Effects of gender related to career plans: F vs M** | | |
| **Factor** |  | | **Odds Ratio** | | **95% Confidence Interval** |
|  | Hispanic | | 0.888 | | 0.481 - 1.641 |
|  | Paid primarily through loans | | 1.276 | | 0.754 - 2.157 |
|  | Sector: Private Practice | | 0.904 | | 0.656 - 1.245 |
|  | **Intention: Education** | | **1.574** | | **1.056 - 2.347** |
|  | Intend: Translational Research | | 0.832 | | 0.627 - 1.104 |
|  | Spec: Internal Medicine | | 0.774 | | 0.586 - 1.023 |
|  | **Spec: Surgery** | | **0.626** | | **0.41 - 0.956** |
|  | Spec: Radiology | | 0.797 | | 0.43 - 1.477 |
|  | Spec: Emergency Med. | | 0.896 | | 0.516 - 1.553 |
|  | **Spec: Family Medicine** | | **2.029** | | **1.008 - 4.083** |
|  | Predict eldercare after res | | 0.941 | | 0.704 - 1.258 |
|  | Obs: Balance clin. res. ed. | | 1.227 | | 0.909 - 1.657 |
|  | **Career Factor: Community Svc** | | **3.886** | | **1.41 - 10.708** |
|  | Can identify mentor | | 0.864 | | 0.523 - 1.426 |
|  | |  | |  |  |
|  | |  | |  |  |

| Supplemental Table 10 | | |  | |  | | |  | |  |  |
| --- | --- | --- | --- | --- | --- | --- | --- | --- | --- | --- | --- |
|  |  | | **MD-PhD, n (%)** | | **MD and MD-RI, n (%)** | | | **P Value** |  |  |  |
| Age category, years | | |  | |  | | |  |  |  |  |
|  | ≤18 | | 2 (0.2%) | | 2 (0.06%) | | | <0.0001 |  |  |  |
|  | 19-24 | | 287 (33.8%) | | 1,566 (47.5%) | | |  |  |  |  |
|  | 25-30 | | 481 (56.7%) | | 1,540 (46.8%) | | |  |  |  |  |
|  | 31-35 | | 74 (8.7%) | | 144 (4.4%) | | |  |  |  |  |
|  | 36+ | | 5 (0.6%) | | 42 (1.3%) | | |  |  |  |  |
| ^1^ = Respondents could select all applicable choices, will not sum to 100% | | | | |  | | |  | |  |  |
|  | |  | |  | |  |  |  |  |  |  |
